# A Time of Great Change: How Parents, Friends, and Classmates Shape Adolescents’ Attitudes towards the Gender Division of Labor

**DOI:** 10.1007/s10964-023-01799-2

**Published:** 2023-07-04

**Authors:** Laia Sánchez Guerrero, Pia S. Schober, Maaike van der Vleuten

**Affiliations:** 1 Department of Sociology, University of Tübingen, 72074 Tübingen, Germany; 2 Department of Sociology, Universitat Autònoma de Barcelona, 08193 Bellaterra (Cerdanyola del Vallès), Spain; 3 Swedish Institute for Social Research (SOFI), University of Stockholm, 106 91 Stockholm, Sweden

**Keywords:** Gender socialization, Gender beliefs, Gender ideologies, Adolescence, Parents, Peers

## Abstract

Parents are crucial in the construction of their children’s attitudes towards the gender division of labor. However, little is known about the extent to which parents’ influences on their children’s attitudes weaken in favor of peers during adolescence. This study explores how gender beliefs of parents, friends, and classmates shape adolescents’ attitudes towards the gender division of labor in Sweden, Germany, England, and the Netherlands. It extends previous research which predominantly examined parent-child transmission. The analysis draws on 4645 children (at wave 1: *M*_age_ = 14.9, SD_age_ = 0.67, females = 50%) of the Children of Immigrants Longitudinal Survey in Four European Countries. Regression analyses of within-person changes in attitudes show that adolescents on average become more egalitarian from age 15 to 16 and significantly adapt their own beliefs to those of their parents, friends, and classmates. In cases of opposing beliefs, adolescents tended to adapt more strongly to whoever held more egalitarian views, possibly aligning with more widespread norms of egalitarianism. The findings show great similarity in adaptation processes across countries and align well with a multi-layered conceptualization of gender as a social structure that shapes gender attitudes.

## Introduction

Adolescence is a dynamic period in the life course in terms of changing gender beliefs. Studying the development of adolescents’ attitudes towards the gender division of labor is important, as they have been shown to predict gendered outcomes among adults in educational aspirations and subject choices (McDaniel, 2010; van der Vleuten et al., 2016), in the division of housework (Platt & Polavieja, 2016) and childcare (Gaunt, 2006), as well as in the labor market (Cunningham et al., 2005; Davis & Greenstein, 2009). These education and labor market outcomes have significant long-term consequences in terms of lower wages and life-time earnings as well as higher poverty risk for women (Bettio et al., 2013; Sigle-Rushton & Waldfogel, 2007) and a restricted ability to combine work and family for men, especially in male-dominated jobs (Grönlund & Öun, 2018; Taylor, 2010). Studies exploring how gender beliefs develop across childhood and adolescence have predominantly examined parent-child transmission (Halimi et al., 2016; Halpern & Perry-Jenkins, 2016). To-date, a small number of studies have also examined influences of the sex composition of peer groups and of peer pressure, but mostly among preschool and elementary school children (for reviews, see Kågesten et al., 2016; Witt, 2000). Such peer influences are likely to become even more influential during adolescence, when individuals start to detach themselves from parental and family influences and increasingly experience new social contexts together with peers (Golshirazian et al., 2015). This study extends existing research by exploring parents and peers together as gender socialization agents during adolescence. Applying a conceptual framework that combines sociological and psychological theories, this research explores the extent to which adolescents adapt their attitudes towards the gender division of labor to reduce discrepancies with those of parents, friends, and classmates and which actors’ attitudes towards the gender division of labor are more relevant in case of diverging beliefs.

### Gender Socialization During Adolescence

Multiple developmental transitions take place during adolescence that may impact gender socialization. First, the onset of puberty goes hand in hand with visible physical changes that make gender more salient for adolescents’ identities as well as in their interactions with others. As a consequence, adolescent bodies are more visibly tied to sex categories and may be increasingly subject to societal gender expectations. Second, adolescents are increasingly able to think abstractly and reflect on themselves and on complex problems (Keating, 1990). Simultaneously, as they advance in their education, they are more likely to be exposed to feminist ideas (Bolzendahl & Myers, 2004; Eccles et al., 2003). Both cognitive development and exposure mechanisms increase the likelihood that they begin reflecting on gender inequality and gendered practices beyond what they have observed from their parents so far. In line with this reasoning, existing studies reported significant increases in gender role flexibility for children from age 10 to 17 in the United States (Katz & Ksansnak, 1994) as well as in egalitarian beliefs for young men and women between ages 15 and 20 in Germany (Wilhelm et al., 2022).

Based on a sociological understanding of gender as a social structure, it is assumed that adolescents’ attitudes towards the gender division of labor are shaped by individual, interactional, and institutional dimensions of societies (Risman, 2004). At the individual level, childhood socialization and gendered parental practices are important influences. Adolescents’ attitudes towards the gender division of labor also vary depending on how gender is reproduced and critically reflected on in every-day interactions, such as with parents and peers at school (West & Zimmerman, 1987). Furthermore, they are shaped by the broader gender culture adolescents are exposed to, such as societal beliefs and their institutional representations (McHale et al., 2003; Risman, 2004). Social-cognitive theory of gender development identified three modes of transmission—modeling, enactive experience, and direct tuition (Bussey & Bandura, 1999). First, modeling implies that children learn about gender norms by observing parents and peers, because a great deal of “gender-linked information is exemplified by models in one’s immediate environment” (Bussey & Bandura, 1999, p. 685). For adolescent’s attitudes towards the gender division of labor, parents’ role modeling in terms of the division of paid and unpaid labor in the family might be particularly relevant. Maternal employment and greater father involvement in domestic work have largely been found to significantly predict more egalitarian beliefs among children in Western countries (Cano & Hofmeister, 2022; Platt & Polavieja, 2016), albeit with some exceptions (Evertsson, 2006). Enactive experience and direct tuition—the other two modes of transmission—refer to social sanctions or rewards and explicit instruction about gender-appropriate behavior in diverse situations. Evidence suggests that parents’ own attitudes permeate their parenting (Antill et al., 2003), and ample research has pointed to associations between parents’ gender beliefs and those of their children (e.g., Cunningham, 2001; Min et al., 2012; Perales et al., 2021; Platt & Polavieja, 2016; Tenenbaum & Leaper, 2002). Most of these studies, however, did not take a dynamic perspective on attitude development across childhood and youth.

A few studies from the United States (US) that considered the longitudinal development across adolescence found that parental attitudes towards the gender division of labor significantly correlated with children’s gender beliefs in middle childhood (around age 10 to 12), but were not associated with subsequent changes in children’s gender beliefs during adolescence (Davis, 2007; Halimi et al., 2021). Hence, they speculated that young people’s other environmental influences, including peers, may be particularly important in adolescence. Due to reduced adult supervision in free-time activities, adolescents rely increasingly on peers for guidance and protection as they navigate novel contexts where peers—alongside and in place of adults—establish and enforce norms (Laursen & Veenstra, 2021). Peer influences tend to increase the resemblance between friends and peers and enhance intragroup norm adherence (Laursen & Veenstra, 2021). Greater norm adherence reduces the risk of harassment and social exclusion (Bos & Sandfort, 2015; Ewing Lee & Troop-Gordon, 2011).

A pioneering study of gender role flexibility in adolescence found that, adolescents’ perceptions of the gender-typicality of the interests and activities of their closest friend, correlated more strongly with their gender-related preferences, and with tolerance for gender role flexibility in others than the gender flexibility of their parents (Katz & Ksansnak, 1994). Some studies have reported greater similarity in gender- and family-related attitudes among friends than among non-befriended peers, which may point to either value similarity being relevant to selection into friendships or to socialization influences from friends (Campigotto et al., 2022; Smith et al., 2014). Only a small number of studies have explored longitudinal socialization processes, i.e., how friends’ or peers’ gendered beliefs or interests shape individuals’ subsequent belief adaptations or gendered choices. Findings from the Netherlands suggest that friends’ more traditional attitudes towards the division of labor were positively associated with more gender-typical subject choices in post-secondary education for girls (van der Vleuten et al., 2019). Similarly, research on Sweden showed that friends’ gendered subject preferences influenced adolescents’ subject preferences in schools (Raabe et al., 2019). A recent longitudinal US study of 12- to 14-year-olds showed that adolescents who perceived greater pressure to conform to friends´ gender norms experienced a greater shift towards more egalitarian beliefs over time (Halimi et al., 2021).

### Boys and Girls as Self-reflective Agents

In the agentic socio-cognitive development perspective, people are understood as “self-organizing, proactive, self-reflective, and self-regulating” and not as passive reactors to external environments and events (Bussey & Bandura, 1999, p. 691). Children self-select their socialization environments and the weight they give to different actors. Girls are more likely to be motivated to adopt egalitarian gender beliefs, as they may recognize the benefits of fewer constraints and greater opportunities for women which more egalitarian gender structures offer. By contrast, boys may be more likely to adapt to or maintain more traditional beliefs, as they benefit from traditional gender structures. Several studies have pointed to this self-interest perspective on gender belief development as one important driver of the consistently more egalitarian gender beliefs reported by females compared to males across the life course (Bolzendahl & Myers, 2004; Fan & Marini, 2000).

### Institutional and Cultural Context

In line with the perspective of gender as a social structure, state policies at the macro-level have been conceptualized as actively creating and reinforcing gender cultures (Chang, 2000; Grunow & Veltkamp, 2016), and these state policies vary substantially in the four countries of our study. Whereas Sweden has long combined a formal commitment to gender equality with strong provision of support services for working mothers and caring fathers (Goldscheider et al., 2011), England and the Netherlands have implemented equal pay and antidiscrimination laws to provide women and men with equal access to the labor market, but have not actively supported gender equality in the private sphere (Chang, 2000; Grunow & Veltkamp, 2016). In line with the traditional family-centered regime, public policies in West Germany long encouraged women to structure their employment around family obligations (e.g., by taking long leaves after childbirth). Despite a paradigm shift since the mid-2000s (Stahl & Schober, 2018), with greater support for more gender-equal work and care arrangements, the cohort of children in our study who were born in the 1990s were probably still influenced by Germany’s familialist norms at the time. Previous studies have confirmed substantial differences in the degree of rejection of a traditional male breadwinner family model across the four countries (Sánchez Guerrero & Schober, 2021). However, the few existing cross-national studies suggest that intergenerational transmission processes from parents to children and normative influences of classmates seem to take place very similarly across these contexts (Sánchez Guerrero & Schober, 2021; Wuestenenk et al., 2022). Hence, this study does not expect significant differences in the predicted socialization processes but contributes to this literature by exploring possible variations empirically.

Finally, in Western European countries, ethnic minority children were found to hold more traditional attitudes towards the gender division of labor on average than those of the majority population (Sánchez Guerrero & Schober, 2021). The intergenerational transmission of gender beliefs from parents to children is mostly very similar among various groups of immigrants and natives (De Valk, 2008; De Valk & Liefbroer, 2007). Only specific groups display stronger intergenerational stability in attitudes towards the gender division of labor, such as Turkish immigrants in Germany (Diehl et al., 2009; Sánchez Guerrero & Schober, 2021). Part of the reason seems to be a higher level of religiosity and religious participation, including affiliation with Islam (Diehl et al., 2009; Röder, 2014). It is beyond the scope of this article to explore ethnic differences in influences of parents and peers in detail but this study controls for its potential influence in the analyses.

## Current Study

Previous research has shown that parents are influential in shaping their children’s gender belief development. As children reach adolescence, peers tend to gain in importance as socialization agents. Yet, previous studies have often failed to consider both parents’ and peers’ influences during adolescence, focusing on either of them but rarely on both. This study explores how the attitudes of parents, friends, and classmates shape adolescents’ attitudes towards the gender division of paid and unpaid work in England, Germany, the Netherlands, and Sweden. It extends previous research by exploring parents and peers jointly as gender socialization influences on changes in adolescents’ attitudes towards the gender division of labor from about age 15 to 16 in four European countries. The study further distinguishes between the influences of friends in class from those of non-befriended classmates and investigates the interdependence of parent and peer influences in the case of opposing beliefs. Based on the theoretical perspectives and previous empirical results, the study generally assumes that parents’ attitudes towards the gender division of labor strongly influence their children’s until about middle childhood or early adolescence, and that children likely choose friends with similar cultural beliefs. As a result, at the first observation point at age 15, adolescents’ attitudes towards the gender division of labor will be more similar to those of their parents and to those of their friends compared to non-befriended classmates. However, after accounting for this initial similarity, if further differences persist, the study expects continuing influences of parents, friends and classmates, as regular contact provides opportunities for direct discussions of gender roles, role modeling as well as experiencing sanctions for non-conformity or rewards for conformity with gender norms. The following hypotheses, thus, are based on the notion that adolescents will adapt their attitudes towards the gender division of labor over time to become more similar to those of their parents, their friends, and to school class norms, which is likely to aggravate or attenuate the average trend towards more egalitarian attitudes during adolescence. First, adolescent children whose parents, friends, and classmates hold more egalitarian attitudes than themselves at the start will experience a larger increase in egalitarianism over one year compared to adolescents who hold similar beliefs as their parents, friends, and classmates, respectively (Hypothesis 1). Second, as peer influences and norm adherence gain in importance during adolescence, while young people may slowly detach themselves from parental influences, friends and non-befriended classmates are expected to be more influential than parents when parents’ gender beliefs deviate from those of peers (Hypothesis 2). It was also stated that adolescents engage in discussions and reflections on gender beliefs with friends, and such discussions are more likely to affect their own beliefs due to the greater relevance and closeness of the relationship than with non-befriended classmates. As a result, the current study expects reciprocal adaptation processes among friends over time. Specifically, greater increases in egalitarianism among friends will be associated with a larger increase in egalitarianism among adolescents over one year (Hypothesis 3). As class compositions changed substantially over the observation period, this study focuses on adaptations over time among friends rather than among non-befriended classmates, as the former relationships are more likely to persist over time. Furthermore, because girls might benefit more from egalitarian beliefs, whereas boys might benefit more from traditional beliefs, the study predicts adaptation processes to vary between boys and girls. Specifically, girls whose parents, friends, and classmates hold more egalitarian attitudes than themselves, or whose friends become more egalitarian, exhibit a larger change towards egalitarianism or a smaller change towards traditionalism than boys (Hypothesis 4). Further, boys whose parents, friends, and classmates hold more traditional beliefs than themselves, or whose friends become more traditional, exhibit a larger change towards more traditional beliefs or a smaller change towards egalitarianism than girls (Hypothesis 5).

## Methods

### Data

The analysis is based on the first two waves of the Children of Immigrants Longitudinal Survey in Four European Countries (CILS4EU, Kalter et al., 2016). In 2010, about 500 schools and over 18,000 pupils in the eighth grade (about 14 years old) were sampled, alongside one of their parents, in England, Germany, the Netherlands, and Sweden. A stratified sampling strategy was used. First, schools were invited to participate and large schools and schools with a larger share of immigrant children were more likely to be invited. Non-responding schools were replaced with other similar schools, and the school response rate after replacement amounted to 83.2%. At least two classes of 14-year-olds were randomly selected and all students in these classes were asked to participate (student response rate was 84.7%). Children filled in a self-guided questionnaire at school and received a questionnaire and a pre-stamped envelope for one of their parents to fill in. The parental response rate was 62% on average, but differed across countries (England = 36.8%, Germany = 78%, the Netherlands = 74.4%, and Sweden = 58.9%) (CILS4EU, 2016b). Given participation in the first wave, the overall student participation rate in wave 2 was 76.6% in England, 82.7% in Germany, 77.5% in the Netherlands, and 81.8% in Sweden (CILS4EU, 2016a).

In total, 18,716 adolescents in 480 schools and 10,663 parents filled in the questionnaires in wave 1 (CILS4EU, 2016b). Among those, 14,939 students in 425 schools also participated in wave 2 (CILS4EU, 2016a). Students were surveyed both in and outside the school in all countries but Sweden, where only in-school surveys were conducted. Students were asked who their best friends in class are (with a maximum of 5), allowing us to separate the impact of the wider classroom from that of friends in class. Parents were only surveyed in wave 1, meaning this study cannot assess how their changes in attitudes towards the gender division of labor (AGDL) are associated with changes in their children’s AGDL. However, the AGDL of adults tend to be more stable than those of children and adolescents, and such changes are mainly driven by major life course transitions and exposure to new contexts, which is less likely for the large majority of parents in our sample who lived in the destination country for over 10 years (Alwin & Scott, 1996; Schober & Scott, 2012).

### Sample Selection and Nonresponse

The analytical sample incorporates all adolescents for whom information on their own and their friends’ attitudes towards the gender division of labor was available in waves 1 and 2, and whose parents and classmates reported their AGDL at wave 1. Of the people who participated in waves 1 and 2, about 44% of the sample was missing because their parents did not fill in the questionnaire (see sensitivity analyses for results excluding parents). Item non-response for the questions used to create the attitudes towards the gender division of labor measure was small (3% at wave 1, 1% at wave 2, and 5% for parents). About 8% of the sample were dropped because the adolescent did not nominate any friends with a valid response on attitudes towards the gender division of labor. Additional analyses show that the latter subsample of adolescents did not differ in their AGDL but was more likely to have an immigrant background and less educated parents than those with full information.

Ultimately, there is information on 4645 adolescents as well as one of their parents (82% mothers), and their friends in class (3.8 friends with AGDL available on average). More precisely, complete information is available for 682 adolescents in England, 1599 in Germany, 1158 in the Netherlands, and 1206 in Sweden. In total, these adolescents are nested in 406 schools and 750 classrooms. To account for the nesting structure of students in classrooms in schools, the estimated standard errors were clustered at the school level. Moreover, the senate weights provided by CILS4EU were used, which correct both for wave nonresponse and give an equal weight to each country in our models (as if the sample in each country was 500 individuals) (for more detailed information, see CILS4EU, 2016b). Thus, once weighted, the data should be representative of the demographic characteristics of the average student population in the four countries.

### Measures

#### Changes in attitudes towards the gender division of labor

The adolescent gender attitude measure drew on four items regarding who should do the following four tasks in a family: (a) Take care of children, (b) cook, (c) clean the house, and (d) earn money. Participants chose one out of three answer categories (1) mostly the man, (2) mostly the woman, and (3) both about the same. The original variables were recoded so higher values would indicate a less traditional distribution of paid and unpaid labor. A polychoric factor analysis (alpha = 0.71) was applied to build a factor, with higher values indicating less traditional AGDL (values in the analytical sample ranged from 1.09 to 3.3). Subtracting the factor value in wave 1 from the value in wave 2 resulted in the dependent variable *change in attitudes towards the gender division of labor*, in which higher values indicate a change towards less traditional, or more egalitarian attitudes. To facilitate interpretation, the variable was z-standardized for the analyses.

#### Difference between adolescent’s own and parent’s attitudes towards the gender division of labor

The parental gender attitude factor was created based on the same four items and following the same approach as for adolescents’ AGDL (alpha = 0.74). To test for measurement invariance of the latent concept of attitudes towards the gender division of labor for parents and children, five models with various parameter constraints were estimated and their model fits compared. As the Chi2 Test is sensitive to the sample size and can lead to dismiss good fitting models in large samples like the one in these models, the Comparative Fit Index (CFI), Tucker-Lewis Index (TLI), and the Root Mean Squared Error of Approximation (RMSEA) are taken as indicators and shown in Table A1 in the Supplementary Material. The results of the goodness of fit indices suggest that metric invariance (Model 2) and strong invariance (Model 3) are met (CFI > 0.95; TLI > 0.95; RMSEA < 0.05), with strict invariance being only partially supported by the tests (Model 4: CFI > 0.90; TLI > 0.90; RMSEA > 0.08 and <0.1; Model 5: CFI > 0.90; TLI > 0.90). Separate Wald tests suggested that two of the four the loadings differed significantly between parents and children. As these results overall point to at least partial invariance, this study follows most previous studies that uses the same measures available for parents and children (e.g., Carlson & Knoester, 2011; Kretschmer, 2018; Perales et al., 2021) and assigned the same factor loadings to parents’ and children’s items to be facilitate comparability between attitudes of parents and children. To create the difference in gender attitudes between parents and adolescents, the former was subtracted from the latter (values range from −1.3 to 1.4). To facilitate interpretation, a categorical version of the resulting measure distinguishes between dyads, in which (1) the parent is more traditional than the adolescent (difference value < −0.1); (2) the attitudes of parent and adolescent are similar (difference values range from −0.1 to 0.1); and (3) the parent is more egalitarian than the adolescent (difference value > 0.1).

#### Difference between the adolescent’s own and friends’ attitudes towards the gender division of labor

First, the mean AGDL at wave 1 of the classmates nominated as friends by the adolescent was computed. Then, the average gender attitude of the friend group was deducted from that of the adolescent (values in our analytical sample range from −1.2 to 1.6). To facilitate interpretation, the resulting measure was recoded into three categories based on whether: (1) the friend group is on average more traditional than the adolescent (difference value < −0.1); (2) the mean AGDL of the friend group and the adolescent are similar (difference value −0.1 to 0.1); and (3) the mean AGDL of the friend group is more egalitarian than the adolescent (difference value > 0.1). To ensure a suitable temporal sequence and comparability of the effects of friends and parents, this variable is also based on the first wave.

#### Difference between the adolescents’ own and non-befriended classmates’ attitudes towards the gender division of labor

First, the mean gender attitude score among non-befriended classmates, excluding the adolescent themself and their friends in wave 1, was computed. Then, the mean gender attitude of the non-befriended classmates was deducted from that of the adolescent in wave 1 (values in our analytical sample range from −1.1 to 1.5) and grouped it into the following categories: (1) the class is on average more traditional than the adolescent (difference value < −0.1); (2) the AGDL of the class and the adolescent are similar (difference value from −0.1 to 0.1); and (3) the class-average gender attitude is more egalitarian than that of the adolescent (difference value > −0.1).

#### Change in gender attitude towards the gender division of labor in the friend group

The mean change in AGDL of nominated friends from wave 1 to wave 2 (the values in our analytical sample range from −1.9 to 1.4) was calculated. Then, the resulting variable was recoded into three categories: (1) friends become more traditional (values < 0); (2) friends do not change (value equals 0); and (3) friends become less traditional (values > 0).

#### Sex of the adolescent

The binary sex categories self-reported by children (girls = 1) were considered. Unfortunately, CILS4EU did not include further measures to distinguish birth-assigned sex and gender identity or other more nuanced measures of gender (role) identities.

### Control Variables

#### Age and age change

The “age” of the adolescent at wave 1 was included in the analysis. Further, “age change” accounts for the change from the first to the second interview, as some adolescents were interviewed at a later stage in the second wave.

#### Immigrant status, religiosity, and religious denomination

A control for the immigrant status of the adolescents and their friends, as well as the adolescents’ religiosity, and religious denomination at wave 1, was included in the analysis. Adolescents were categorized as “immigrant” if they were first- or second-generation immigrants. The latter consist of native-born adolescents with at least one parent born abroad. The third generation was considered as “native”, as they tend to report very similar social attitudes and practices as them (Heinrich-Böll-Stiftung, 2010; Logan & Shin, 2012). Further, a religiosity factor using polychoric factor analysis (alpha = 0.83) was built based on three items: (a) how often the adolescent visits a religious meeting place, (b) how often the adolescent prays, and (c) how important religion is to him/her. This variable was z-standardized for the regression analyses. Finally, a measure of religious denomination distinguished between (1) no religion, (2) non-Muslim (including Christianity, Judaism, Buddhism, Hinduism, Sikhism, and other religions), and (3) Muslim, was included in the analysis.

#### Same-sex parent and sex composition of friend group and class

A control for whether the reporting parent was of the same sex as the adolescent (1) or not (0) was added. It is worth noting that about 82% of our analytical sample are mothers. Thus, while only 18% of males have a same-sex parent responding, 84% of girls have a same-sex parent as the respondent. As only a few adolescents had other-sex friends (25%), a dummy variable for whether all friends are of the same-sex (1) or not (0) was also included. Finally, a control accounting for the share of same-sex classmates, measured as the percentage of students of the same sex in a class excluding the adolescent him- or herself and his or her nominated friends, was included in the models.

#### Maternal employment status

A dummy variable accounting for whether the mother is employed (1) or not (0) was included. Unfortunately, there is no information about their working hours or part- or full-time employment.

#### Number of friends

A control for how many friends in class the adolescents nominated (max 5) was added to the analysis.

#### Parental educational level

The model includes a control for the highest educational level of either the mother or the father, as reported by the parent and measured in three categories: (1) less than upper secondary school; (2) upper secondary school; and (3) tertiary education.

#### Percentage of immigrants in class and percentage of immigrants in school

As the sampling design oversampled schools with a high share of immigrant children, the model includes a control for the share of immigrants in the classroom, measured with a continuous variable, and another control accounting for the share of immigrants in the school, captured in five categories: (1) 0–10%, (2) 10–30%, (3) 30–60% and (4) 60–100%, and (5) independent schools. The latter are schools for which this information is not available (3% of the schools in our sample). The share of immigrants in class is also a proxy control for tracking in Germany and the Netherlands, as immigrant and native students in these countries tend to congregate in different tracks.

#### Receiving country

To account for the country of residence and possible differences due to both social and institutional frameworks, a control for whether students live in England, Germany, Sweden, and the Netherlands is added.

### Analytical Strategy

The analysis used ordinary least squares (OLS) regression with the dependent variable measuring within-person changes in AGDL from wave 1 to wave 2 (standard errors clustered at the school level). One of the challenges in studying peer influence on beliefs lies in reciprocal effects that children are likely to have on each other. In this study, this challenge was addressed by examining these effects longitudinally, seeking to explain changes in adolescents’ AGDL, and using lagged measures of differences between children’s own beliefs relative to their parents, friends, and classmates, respectively. Moreover, as friends’ attitudes and influences may be endogenous to the attitudes of the adolescents themselves, an instrumental variable (IV) regression model was estimated. More precisely, the difference between the attitudes of the parents and friends vis-a-vis children’s own beliefs in wave 1 were used as an IV. The results from the IV model can be found in the sensitivity analysis section.

To test Hypothesis 1, which expected adolescents to experience larger growth towards egalitarianism if parents, friends, and classmates hold more egalitarian beliefs than themselves at the start, the regression models include three independent variables regarding whether parents, friends, and classmates, respectively, hold more egalitarian, similar, or more traditional beliefs than the respective student at wave 1. Changes in the beliefs of friends across the two waves were considered to test whether greater increases in egalitarianism among friends are associated with corresponding changes in adolescents’ own attitudes (Hypothesis 3). To test for stronger influences of friends and classmates than parents in the case of diverging beliefs (Hypothesis 2), a second set of models includes interaction terms of the difference in attitudes between adolescents and their parents with the difference in beliefs between them and their friends and classmates, respectively. Thirdly, these four variables were interacted with the adolescent’s sex to test for differences in adaptation effects between male and female adolescents (Hypotheses 4 and 5). To make the tables parsimonious, the effects of the less important control variables were included in Appendix A, Tables A2 and A3 in the Supplementary Material.

## Results

### Descriptive Results

Table 1 shows descriptive statistics for the key variables in the analyses as well as some key variables used to create them. With respect to the dependent variable, about 24% of adolescents become more traditional (change in attitudes towards the gender division of labor (AGDL) < −0.1), while 45% do not change their attitudes towards the division of labor (change in AGDL between 0.1 and −0.1), and 31% become more egalitarian (change in AGDL > 0.1). With respect to the independent variables, Table 1 shows that adolescents have the largest similarities in AGDL with their parents (43%), followed by their friends (23%) and the least with their classmates (16%). This greater similarity is in line with arguments that parents represent important socialization agents throughout childhood. Only 21% of the adolescents in the sample are more egalitarian than their parents, but 42% and 48% of the adolescents are more egalitarian than their friends and classmates, respectively. Lastly, in around 35–36% of cases, parents, friends, and classmates are more traditional than the adolescents themselves.

**Table 1:** Descriptive statistics

Continuous variables	*M*(SD)	Min	Max
Change in Attitudes Towards the Gender Division of Labor (AGDL)	0.037 (0.352)	−1.642	1.381
AGDL in wave 1	1.836 (0.395)	1.094	3.283
AGDL in wave 2	1.874 (0.380)	1.094	3.283
AGDL difference with parent	−0.098 (0.427)	−1.309	1.382
AGDL difference with friends	0.002 (0.403)	−1.201	1.642
AGDL difference with classmates	0.012 (0.389)	−1.089	1.454
Change in AGDL of friends	0.039 (0.268)	−1.916	1.382
Age at wave 1	14.87 (0.671)	13	18
Age change	0.868 (0.491)	0	2
Share of same-sex classmates	17.05 (7.55)	0	63
Number of friends	3.747 (1.181)	1	5
Share of immigrants in class	20.97 (17.40)	0	100
Religiosity	1.913 (0.962)	1.013	5.183

Categorical variables	% (*N*)		
Change in AGDL			
Become more traditional	24 (1105)		
Do not change	45 (2095)		
Become more egalitarian	31 (1445)		
AGDL difference with parent			
Parent is more traditional	21 (968)		
Parent is similar	43 (1999)		
Parent is more egalitarian	36 (1678)		
AGDL difference with friends			
Friends are more traditional	42 (1957)		
Friends are similar	23 (1046)		
Friends are more egalitarian	35 (1642)		
AGDL difference with classmates			
Classmates are more egalitarian	48 (2244)		
Classmates are similar	16 (721)		
Classmates are more egalitarian	36 (1680)		
AGDL change of friends			
Friends become more traditional	30 (1393)		
No change	26 (1193)		
Friends become more egalitarian	44 (2059)		
Female (ref. Male)	50 (2306)		
Same-sex parent	51 (2371)		
All same-sex friends	75 (3485)		
First- or second-generation immigrant	16 (740)		
Share of immigrants in school			
0–10%	54 (2515)		
10–30%	32 (1485)		
30–60%	9 (404)		
60–100%	2 (92)		
Independent schools	3 (149)		
Mother is employed	85 (3967)		
Parental education level			
Below upper secondary	39 (1806)		
Upper Secondary	32 (1464)		
University degree	30 (1375)		
Religious denomination			
No religion	35 (1628)		
Non-Muslim	62 (2868)		
Muslim	3 (149)		
Country			
England	15 (693)		
Germany	32 (1488)		
The Netherlands	30 (1399)		
Sweden	21 (1065)		

Senate weights applied

**Fig. 1:**
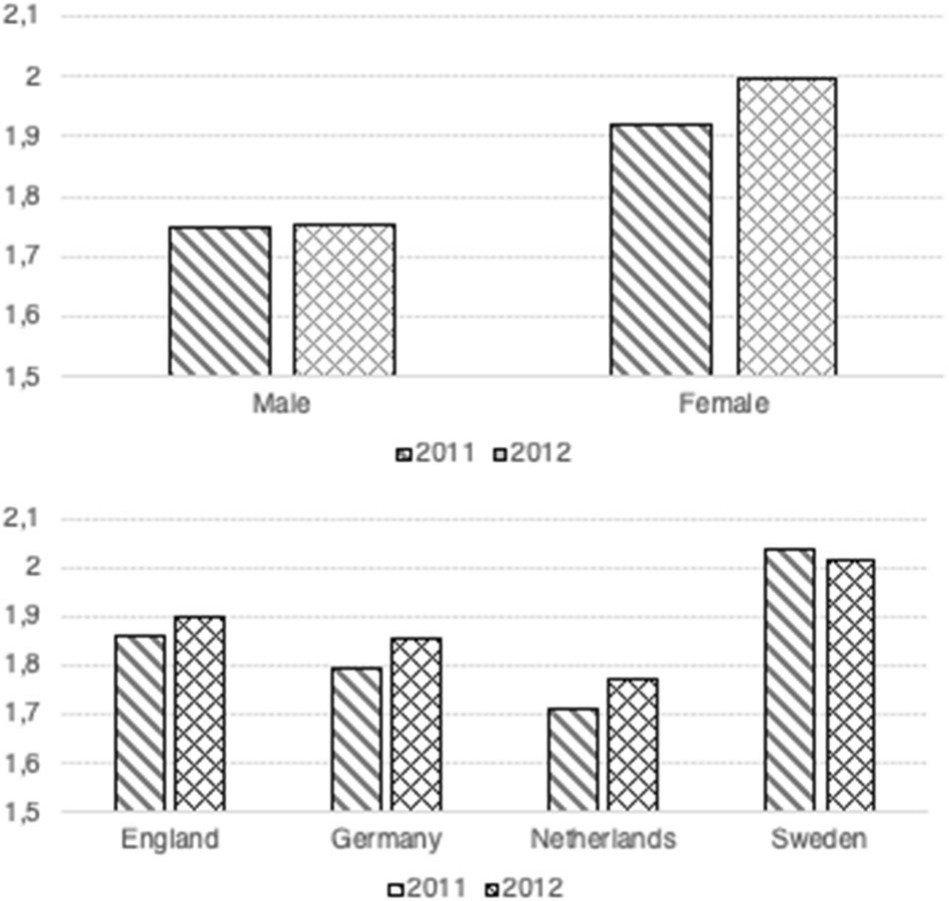
Mean attitudes towards the division of labor in waves 1 (2011) and 2 (2012), by adolescent’s sex (top) and country (bottom)

Figure 1 shows the attitudes towards the gender division of labor in waves 1 and 2 for boys and girls (top) and in different countries (bottom). First, girls appear to report more egalitarian attitudes than boys in both waves and also progress more towards the gender egalitarianism between waves 1 and 2. The bottom panel shows that adolescents in Sweden are the most egalitarian, followed by England and Germany. Adolescents in the Netherlands report the most traditional attitudes. Whereas the change in attitudes of adolescents in Sweden is not statistically significant, adolescents in the other countries become significantly less traditional between waves 1 and 2.

Pearson’s pairwise correlations (see Table A5) show a negative correlation between adolescents’ AGDL at wave 1 with the dependent variable “Change in attitudes towards the gender division of labor” (−0.51). Hence, those who are more egalitarian at wave 1 change less towards egalitarianism in a one-year time span, possibly because for them, the scale leaves less room for further change in the same direction (ceiling effect). Nonetheless, AGDL at wave 2 positively correlate with attitudes at wave 1 (0.44). Hence, those who were more egalitarian at wave 1 still advance towards egalitarianism, just at a slower pace. The correlation is larger for boys (0.48) than for girls (0.37) probably because they have more room to grow between waves 1 and 2 (as shown in Fig. 1).

### Explanatory Results

Table 2 shows the regression models testing Hypotheses 1 to 3. In line with the descriptive results, adolescents on average become significantly more egalitarian from 9th to 10th grade (*b* = 0.147, *p* < 0.001). Furthermore, the older the adolescent is in wave 1, the larger the change towards egalitarianism (*b* = 0.080, *p* < 0.01). Model 1 includes the variables measuring the differences between the adolescents’ attitudes towards the gender division of labor and those of their parents, friends, and classmates, respectively, to test whether adolescents whose parents, friends and classmates hold more egalitarian beliefs at the start will experience larger increases in egalitarianism over the year (Hypothesis 1). In support of Hypothesis 1, Model 1 shows that when parents (*b* = 0.153, *p* < 0.001), friends (*b* = 0.333, *p* < 0.001) or classmates (*b* = 0.251, *p* < 0.001) are more egalitarian than the adolescent themself at wave 1, compared to when they are similar in AGDL, adolescents experience a larger increase in egalitarianism between waves 1 and 2. Similarly, adolescents experience a smaller increase in egalitarianism or a larger increase in traditionalism across the year if their parents (*b* = −0.254, *p* < 0.001), friends (*b* = −0.241, *p* < 0.001) or classmates (*b* = −0.176, *p* < 0.01) are more traditional than themself in wave 1, compared to when they are similar in AGDL at wave 1. Overall, friends appear to be slightly more important, but there are no big differences in the importance of these three groups of actors, and the associations found are of moderate strength, between 15% and 33% of a standard deviation, for all three groups.

**Table 2:** OLS regression for change in adolescents’ attitudes towards the gender division of labor (AGDL) (*N* = 4645)

Variables	Model 1	Model 2	Model 3
AGDL difference with parent (Ref. Similar)			
Parent is more traditional than the adolescent	−0.254*** (0.060)	0.018 (0.079)	0.056 (0.123)
Parent is more egalitarian than the adolescent	0.153** (0.056)	0.268** (0.096)	0.242* (0.109)
AGDL difference with friends (Ref. Similar)			
Friends are more traditional than the adolescent	−0.241*** (0.064)	−0.096 (0.061)	−0.233*** (0.068)
Friends are more egalitarian than the adolescent	0.333*** (0.053)	0.377*** (0.117)	0.032*** (0.060)
AGDL difference with classmates (Ref. Similar)			
Classmates are more traditional than the adolescent	−0.176** (0.064)	−0.176** (0.056)	−0.059 (0.096)
Classmates are more egalitarian than the adolescent	0.251*** (0.076)	0.245*** (0.070)	0.395*** (0.116)
Change in friends’ AGDL (Ref. No change)			
Friend group becomes more traditional	0.030 (0.070)	0.023 (0.067)	0.028 (0.096)
Friend group becomes less traditional	0.096* (0.043)	0.093 (0.044)	0.090** (0.045)
Difference with friends * Difference with parent (Ref. Similar)			
Friends are more traditional & parent is more traditional		−0.367*** (0.078)	
Friends are more traditional & parent is more egalitarian		−0.309** (0.125)	
Friends are more egalitarian & parent is more traditional		−0.304** (0.117)	
Friends are more egalitarian & parent is more egalitarian		−0.333 (0.140)	
Difference with classmates * Difference with parent (Ref. Similar)			
Classmates are more traditional & parent is more traditional			−0.327** (0.116)
Classmates are more traditional & parent is more egalitarian			−0.165 (0.137)
Classmates are more egalitarian & parent is more traditional			−0.471*** (0.126)
Classmates are more egalitarian & parent is more egalitarian		−0.088 (0.115)	
Age at wave 1	0.080* (0.032)	0.033* (0.022)	0.081* (0.032)
Difference in age between waves	0.147*** (0.044)	0.141*** (0.041)	0.139*** (0.053)
Female (Ref. Male)	0.369*** (0.061)	0.360*** (0.058)	0.369*** (0.062)
Immigrant status (Ref. Native/3rd generation)	−0.042 (0.041)	−0.043 (0.040)	−0.046 (0.041)
Country of residence (ref. Sweden)			
England	0.127 (0.078)	0.113 (0.074)	0.118 (0.077)
Germany	0.133* (0.058)	0.124* (0.060)	0.124* (0.058)
The Netherlands	0.146 (0.075)	0.146 (0.080)	0.134 (0.073)
Constant	−1.602** (0.531)	−1.600*** (0.480)	−1.721*** (0.520)
*R*^2^	0.29	0.29	0.29

All models include additional controls for number of friends, whether all friends are of the same sex, whether responding parent is of the same sex, share of same-sex classmates, parental education, maternal employment status, religious denomination, religiosity, and share of immigrants in school (for results see Table A2)

*p* values: * < 0.05; ** < 0.01; *** < 0.001

**Fig. 2:**
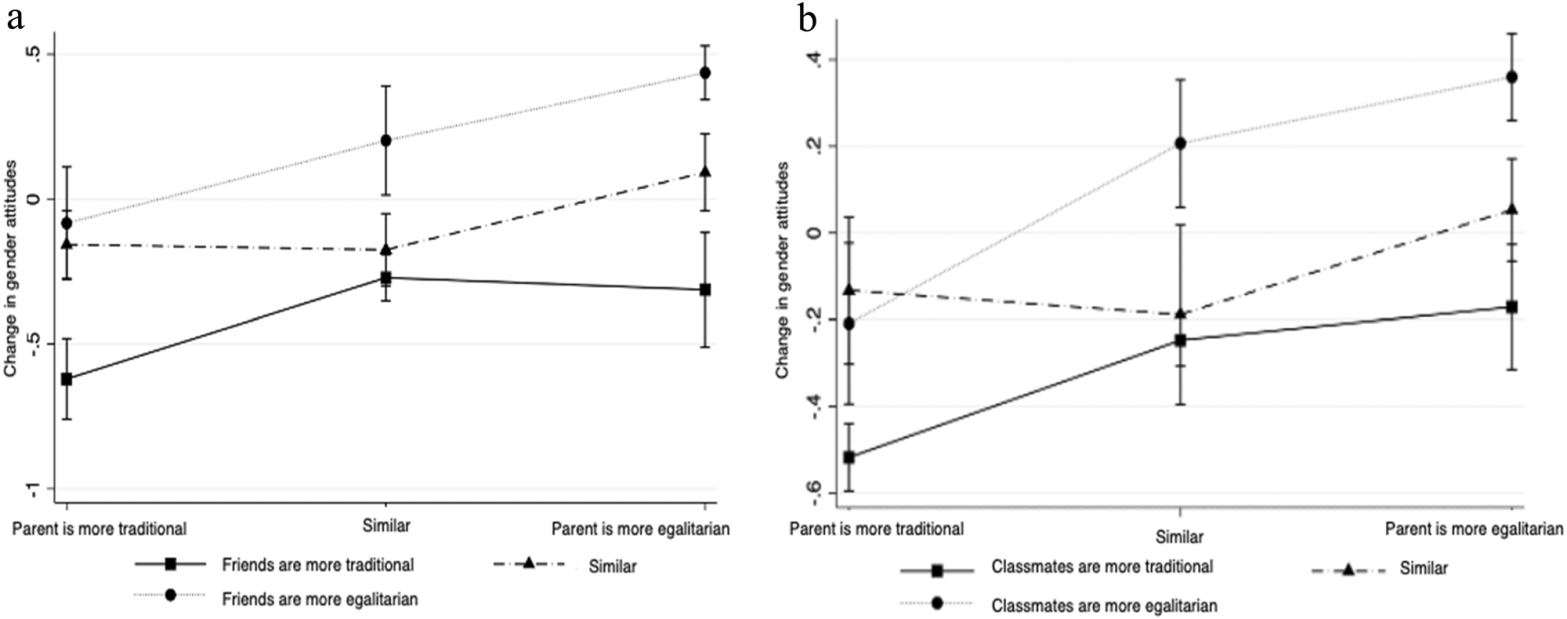
Predictive margins of the interaction between differences between adolescents’ own and friends’ attitudes towards the division of labor (**a**, left) and classmates’ attitudes towards the division of labor (**b**, right) on the change in adolescents’ attitudes towards the division of labor

Models 2 and 3 in Table 2 include interactions between the *difference between the adolescent’s and the parent’s AGDL*, the *difference between the adolescent’s and friends´ AGDL* (Model 2) as well as the *difference between the adolescent’s and classmates´ AGDL* (Model 3) to evaluate Hypothesis 2 that when parents’ AGDL diverge from those held by friends and classmates, adolescents will adapt their AGDL more strongly to those of their friends and classmates than to the attitudes held by their parents. Hypotheses 2 is therefore tested for friend and classmates separately. Model 2 shows significant interactions, which are plotted in Fig. 2 to facilitate interpretation. Not surprisingly, adolescents report the largest increases in egalitarianism when friends as well as parents are more egalitarian (Fig. 2a, light gray line, right dot; 0.377 + 0.268 = 0.645 in Model 2, Table 2). Moreover, when parents as well as friends are more traditional, adolescents experience a strong increase in traditionalism (black solid line, left dot; 0.018–0.096 – 0.367 = −0.455 in Model 2, Table 2). When parents and friends differ in opposing ways from adolescents’ own attitudes towards the gender division of labor, the effects mostly offset each other, resulting in no significant differences to adolescents whose socialization agents hold similar views as themselves. Interestingly, non-linear patterns can only be seen for constellations when adolescents differ only from either their parents or their friends. When they resemble their parents, but their friends hold more egalitarian views, their beliefs become more egalitarian (*b* = 0.377, *p* < 0.001), and a similar change (*b* = 0.268, *p* < 0.01) occurs when they resemble their friends, but their parents hold more egalitarian views. By contrast, when they resemble one of these groups and the other group holds more traditional beliefs, they do not differ in their gender attitude development from those who are similar to both groups (see non-significant main effects of “parent more traditional” and “friends more traditional” in Model 2). This does not lend support to the Hypothesis 2, that friends are more important than parents in the case of differing attitudes.

Model 3 in Table 2 also shows significant interactions, which are plotted in Fig. 2b. These results largely follow the same patterns as those in Fig. 2a and also do not lend support to the hypothesis that classmates are more important than parents when their AGDL diverge (Hypothesis 2 tested for classmates). When parents and classmates differ in opposing ways from adolescents’ own AGDL, the effects largely offset each other, resulting in no significant differences to adolescents whose socialization agents hold similar views as themselves. When adolescents resemble their parents, but their classmates hold more egalitarian views, they change towards more egalitarian beliefs (*b* = 0.395, *p* < 0.001), and a slightly weaker change (*b* = 0.242, *p* < 0.01) occurs when they resemble their classmates, but their parents hold more egalitarian views. By contrast, when they resemble one of these groups and the other group holds more traditional beliefs, they do not differ in their AGDL development from those who are similar to both groups (see non-significant main effects of “parent more traditional” and “classmates more traditional” in Model 2).

To test Hypothesis 3 that greater increases in egalitarianism among friends will be associated with a larger increase in egalitarianism among the adolescents themselves over the year, Model 1 includes the variable *change in friends´ AGDL*. The results suggest that, compared to friends who remain similar in AGDL between waves 1 and 2, having friends who become more egalitarian over time is associated with larger changes towards egalitarianism for adolescents (*b* = 0.096, *p* < 0.05). Changes towards more traditional attitudes among friends are not significantly associated with changes in adolescents’ own AGDL. Hypothesis 3 is therefore only partially supported.

Models 1 to 4 in Table 3 test whether parents, friends or classmates are of different importance for boys and girls. Specifically, it tests whether girls whose parents, friends, and classmates, respectively, hold more egalitarian beliefs than themselves, or whose friends become more egalitarian, exhibit a larger change towards egalitarianism or a smaller change towards traditionalism than boys (Hypothesis 4). And whether boys whose parents, friends, and classmates hold more traditional beliefs than themselves, or whose friends become more traditional, exhibit a larger change towards more traditional beliefs or a smaller change towards egalitarianism than girls (Hypothesis 5). This is tested for parents, friends, and classmates separately before reaching an overall conclusion about Hypotheses 4 and 5.

**Table 3:** OLS for change in adolescents’ attitudes towards the gender division of labor (AGDL) including interactions with adolescent sex (*N* = 4645)

Variables	Model 1	Model 2	Model 3	Model 4
AGDL difference with parent (Ref. Similar)				
Parent is more traditional than the adolescent	−0.330** (0.105)	−0.256*** (0.060)	−0.256*** (0.060)	−0.255*** (0.059)
Parent is more egalitarian than the adolescent	0.009 (0.076)	0.147** (0.057)	0.151** (0.058)	0.153** (0.055)
AGDL difference with friends (Ref. Similar)				
Friends are more traditional than the adolescent	−0.256*** (0.069)	−0.291*** (0.080)	−0.250*** (0.065)	−0.242*** (0.064)
Friends are more egalitarian than the adolescent	0.312*** (0.055)	0.247** (0.084)	0.326*** (0.054)	0.333*** (0.054)
Change in friends’ AGDL (Ref. No change)				
Friend group becomes more traditional	0.027 (0.068)	0.032 (0.072)	0.032 (0.071)	−0.029 (0.084)
Friend group becomes less traditional	0.091* (0.043)	0.100* (0.045)	0.097* (0.044	0.045 (0.072)
AGDL difference with classmates (Ref. Similar)				
Classmates are more traditional than the adolescent	−0.162* (0.067)	−0.167* (0.067)	−0.181* (0.090)	0.175** (0.063)
Classmates are more egalitarian than the adolescent	0.262** (0.082)	0.250** (0.079)	0.196* (0.081)	0.252*** (0.077)
AGDL difference with parent * Female (Ref. Similar/male)				
Parent is more traditional than the adolescent	0.133 (0.107)			
Parent is more egalitarian than the adolescent	0.326** (0.125)			
AGDL difference with friends * Female (Ref. Similar/male)				
Friends are more traditional than the adolescent		0.068 (0.106)		
Friends are more egalitarian than the adolescent		0.175 (0.095)		
Change in friend’s AGDL * female (Ref. No change/male)				
Friend group becomes more traditional				0.196 (0.113)
Friend group becomes less traditional				0.060 (0.080)
Difference with classmates * Female (Ref. Similar/male)				
Classmates are more traditional than the adolescent			0.025 (0.107)	
Classmates are more egalitarian than the adolescent			0.136 (0.116)	
Age at wave 1	0.071* (0.029)	0.079* (0.031)	0.076* (0.031)	0.081* (0.032)
Difference in age between waves	0.138*** (0.040)	0.145*** (0.042)	0.143*** (0.042)	0.149*** (0.043)
Female (Ref. Male)	0.219*** 0.049	0.275** (0.107)	0.307** (0.118)	0.314*** (0.083)
Immigrant background (Ref. Native/3rd generation)	−0.041 (0.040)	−0.044 (0.040)	0.041 (0.041)	−0.041 (0.041)
Country of residence (ref. Sweden)				
England	0.130 (0.078)	0.125 (0.078)	0.125 (0.078)	0.118 (0.078)
Germany	0.132* (0.058)	0.131* (0.058)	0.134* (0.058)	0.127* (0.057)
The Netherlands	0.141 (0.073)	0.143 (0.076)	0.143 (0.074)	0.137 (0.073)
Constant	−1.384*** (0.448)	−1.532** (0.516)	−1.507** (0.510)	−1.599** (0.504)
*R*^2^	0.29	0.29	0.29	0.29

All models include additional controls for number of friends, whether all friends are of the same sex, whether responding parent is of the same sex, share of same-sex classmates, parental education, maternal employment status, religious denomination, religiosity, and share of immigrants in school (for results see Table A3)

*p* values: * < 0.05; ** < 0.01; *** < 0.001

**Fig. 3:**
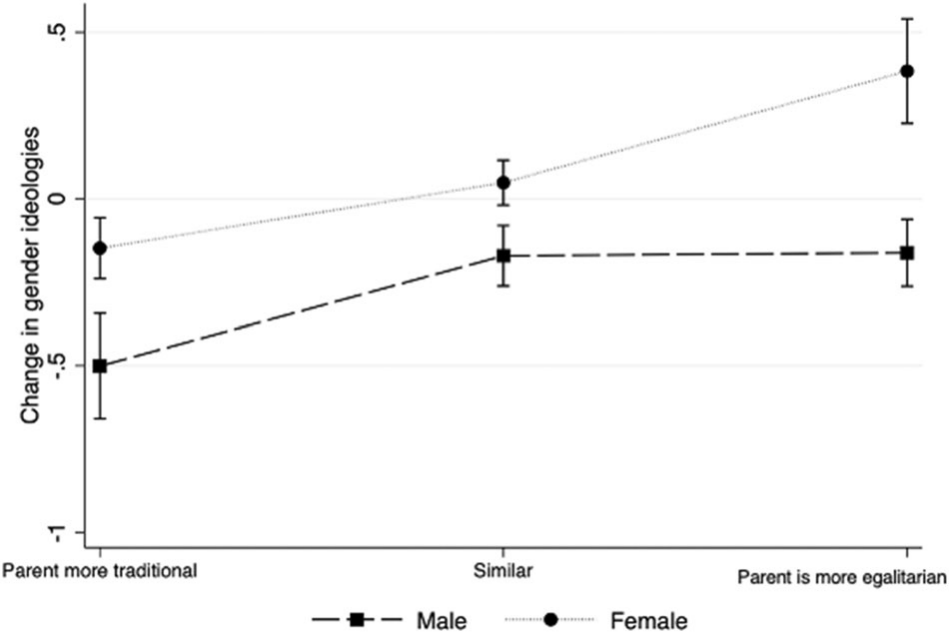
Predictive margins of the interaction of differences between adolescents’ own and parents’ attitudes towards the division of labor with adolescents’ sex on the change in adolescents’ attitudes towards the division of labor

First, it is important to note that girls show a larger increase in egalitarianism than boys (Model 1, Table 2: *b* = 0.369, *p* < 0.001). Model 1 in Table 3 includes an interaction between the *difference between the adolescents’ and their parents’ AGDL* and *sex* to test differences in parental influence between boys and girls (Hypotheses 4 and 5). In line with H4, Model 1 in Table 3 and Fig. 3 shows that girls experience larger changes towards gender egalitarianism than boys when their parents are more egalitarian than the adolescent in wave 1 (*b* = 0.326, *p* < 0.01). Model 1 shows no evidence that boys experience a larger change towards more traditional attitudes or a smaller change towards egalitarianism than girls when their parents are more traditional, leading to the rejection of H5 with respect to parental influences.

Model 2 includes an interaction of the *differences between adolescents’ own and their friends’ AGDL* and *sex* to examine whether girls whose friends hold more egalitarian beliefs than themselves change more strongly towards egalitarianism than boys (H4), as well as whether boys whose friends hold more traditional beliefs than themselves exhibit a larger change towards more traditional beliefs or a smaller change towards egalitarianism than girls (H5). However, the effects are not significantly different for boys and girls, refuting these hypotheses. Following the same logic also for classmates, Model 3 tests H4 and H5 by including an interaction of the *differences between adolescents’ and classmates’ AGDL* and *sex of the child*. Again, the interaction effects do not reach statistical significance, leading to the rejection of both H4 and H5 regarding friends’ and classmates’ influences.

Model 4 includes an interaction between *changes in friends´ attitudes towards the gender division of labor* and *female* to test whether girls whose friends become more egalitarian exhibit a larger change towards egalitarianism than boys (H4), and boys whose friends become more traditional exhibit a larger change towards more traditionalism or a smaller change towards egalitarianism than girls (H5). The effects are not significant, again refuting Hypotheses 4 and 5 for reciprocal influences of friends. Overall, this means that hypothesis 4 is partly supported because there is evidence that girls experience larger changes towards gender egalitarianism than boys when their parents are more egalitarian, but no association was found for friends or classmates. The data does not support hypothesis 5.

### Institutional and Cultural Context

Table A4 in the Supplementary Material tests whether differences with parents, friends or classmates lead to different adaptations in adolescents’ AGDL depending on the country of residence by including country interactions. As expected, there are no substantial country differences in the importance of the actors. The only difference found is that differences between adolescents’ own and their friends’ AGDL are associated with smaller changes towards the gender egalitarianism in the Netherlands compared to Sweden (*b* = −0.514; *p* < 0.001; *b* = −0.388, *p* < 0.001). This implies that the associations between adolescents’ changes in AGDL and the AGDL of their parents, friends, and classmates are on the whole very similar across the four countries.

### Control Variables

Tables A2 and A3 in the Supplementary Material show the effects of all control variables corresponding to the models in Tables 2 and 3 respectively. Apart from age and sex, very few of the control variables show significant associations with changes in adolescents’ AGDL over time. A higher share of same-sex classmates decreases changes towards egalitarianism (*b* = 0.007, *p* < 0.05). Adolescents in schools with a higher share of immigrants (60–100%) change about 15% of a standard deviation more towards egalitarianism compared to students attending a school with a very low share of immigrants (0–10%). Moreover, adolescents in Germany change about 13% of a standard deviation more towards egalitarianism between the two waves than adolescents in Sweden.

### Sensitivity Analyses

The results of all sensitivity analyses are available upon request. First, a test of whether the adaptation processes described in Hypotheses 1 and 3 vary between ethnic minority and majority populations was performed by interacting the four key independent variables (differences in AGDL between adolescents and parents, friends, and classmates, respectively, as well as changes in friends’ AGDL) with immigrant status. No significant differences were found.

Second, gender socialization theories suggest that same-sex parents and same-sex friends may be particularly important for the transmission of AGDL because they are more likely to represent role models (Bussey & Bandura, 1999; Johnson, 1975). Among peers, however, the views of other-sex peers may have equal weight, as they may represent potential dating partners. Additional tests with interaction terms with parent sex and sex compositions of friends and classmates, respectively, showed no statistically significant differences in the attitudinal adaptation processes depending on whether the parent and friends were of the same sex and by sex compositions of classes.

Third, the parents who did not fill in the parent questionnaire (little over 40%) at wave 1 may be a selective group. Hence, additional tests were performed to see whether the results for friends and classmates hold when Models 1 and 2 are rerun for a larger sample of 8453 adolescents, including those without parental reports. The effects of friends and classmates became somewhat stronger, but excluding parents did not lead to any different conclusions with respect to the importance of friends and classmates.

Fourth, it might be hard in small classes to separate the influence of friends and classmates. Therefore, all analyses were run again only for classes with 10 or more participating pupils. This did not affect the conclusions.

Fifth, to test whether the results are sensitive to the cut-off values of ±0.1 points for the measures of differences in AGDL between adolescents and their parents, friends, and classmates, alternative cut-off points of ±0.2 were tested and led to virtually the same results.

Sixth, as adolescents and their parents may interpret the items that were used to create the attitudes towards the gender division of labor differently across the four countries, measurement equivalence was tested by applying structural equation modeling. However, the models presented convergence issues and, therefore, an alternative approach was taken to test the robustness of the results. To test whether the regression results presented in Table 2 are sensitive to constraining the factor loadings to be equal across countries, a set of alternative single-country models were tested including our main independent variables (AGDL differences with parents, friends, classmates and changes in friend’s AGDL) which allowed the factor loadings to vary by country. As Table A6 shows, the substantive results of these single-country models with country-specific factor loadings are very similar to those of single-country models with factor loadings based on the pooled sample.

Seventh, to test whether reverse causality is likely to bias the results for adolescents’ adaptations to their friends’ AGDL, instrumental variable regressions were applied by using the AGDL of friends’ parents as exogeneous predictors. Hence, the assumption is that the AGDL of friends’ parents are (1) related to friends’ attitudes via parental socialization, but (2) do not directly affect the object of analysis: the change in adolescents’ AGDL. The results are shown in Table A7 in the Supplementary Material. The first stage results supported the suitability of this instrument. The second stage of the instrumental variable regressions showed a statistically significant and strong positive effect of more egalitarian friends on an increase in adolescents’ egalitarian attitudes (*b* = 0.542, *p* < 0.001), whereas more traditional friends were not significantly related to changes in adolescents’ AGDL. These results therefore partially confirm the results of Model 1 in Table 2 and suggest that adaptations to more traditional AGDL held by friends may be more subject to bias due to reverse causality.

Lastly, the analyses were run with continuous instead of categorical measures of differences between adolescents and their parents, friends, and classmates, respectively. The associations were similarly or more significant and point in the same direction, but are harder to interpret, as non-linear associations cannot be accounted for.

## Discussion

While there is a growing body of research on parent-child transmission of attitudes towards the gender division of labor, little is known about how peers contribute separately and jointly with parents to adolescent attitudes towards the gender division of labor development. This study investigated the importance of parents, friends, and classmates in shaping adolescents’ attitudes towards the gender division of labor from 9th to 10th grade in Sweden, Germany, England, and the Netherlands. The analysis provided evidence of adolescents systematically adapting their attitudes to those of all three groups of socialization agents that were studied. The associations were of moderate strengths of between 15% and 33% of a standard deviation.

In line with a small number of previous longitudinal studies on adolescents’ gender beliefs in the U.S. (Katz & Ksansnak, 1994) and in Germany (Wilhelm et al., 2022), this study found considerable variation in adolescents’ attitudes mostly towards greater support for a more gender-equal division of labor even over the period of one year from about age 15 to 16. Investigating the relevance of parents, friends and classmates for this time period therefore seems relevant and informative, although ideally future studies should explore longer-term trajectories.

For parents, the analysis found a strong correlation with the attitudes adolescents reported at grade 9, which is in line with previous evidence on the relatively strong transmission of gender beliefs from parents to children (McHale et al., 2003; Tenenbaum & Leaper, 2002). In contrast to two previous studies (Davis, 2007; Halimi et al., 2021), which found that parents’ attitudes towards the gender division of labor did not predict changes in adolescents’ gender beliefs over time, the findings of this study suggest that adolescents continue to align their attitudes over time with those reported by their parent. The differing results may be due to differences in the estimation method and operationalization. Whereas prior studies considered parents’ level of egalitarianism as predictor, this study focuses on differences between parents and children, which are more strongly related to subsequent within-person changes in attitudes towards the gender division of labor.

Interestingly, the results of this study point to a significant difference in this adaptation process between boys and girls. Girls change more strongly towards more egalitarian attitudes when their parents hold more egalitarian views than boys. This is in line with self-interest explanations suggesting that girls are more motivated to adopt egalitarian gender beliefs because they recognize the benefits associated with more egalitarian gender structures (Bolzendahl & Myers, 2004; Bussey & Bandura, 1999). It is also noteworthy that additional tests showed that this difference is not driven by stronger transmission of attitudes towards the gender division of labor among parents and children of the same sex, as no significant interactions with whether the parent was of the same or another sex were found. This adds to the mixed findings of previous studies, many of which also do not find stronger cultural transmission among same-sex parent-child dyads (Dawson et al., 2015; Kulik, 2002; Law & Schober, 2021; van der Vleuten et al., 2018). However, most of the parents in the sample were mothers and the fathers who participated might be a selective group (e.g., spend more time at home, more involved in their child´s education). This could be a reason why this study does not identify different socialization effects between same-sex and opposite-sex parent-child dyads, or between mothers or fathers. Hence, future research should replicate these results with a more balanced number of fathers and mothers and ideally reports from all social parents.

In line with previous studies on gender-typical study choices in the Netherlands (van der Vleuten et al., 2019) and subject preferences in schools in Sweden (Raabe et al., 2019), the results of this study suggest that friends within school classes as well as other classmates are influential socialization agents. The findings provide evidence of adolescents changing their attitudes towards the gender division of labor in such a way as to reduce pre-existing differences with their friends and with non-befriended classmates as well as of conjoint development among adolescents and their friends towards more egalitarian attitudes towards the gender division of labor over time. However, friends adopting more traditional beliefs over time did not predict corresponding changes in adolescents’ own attitudes towards the gender division of labor, meaning that this finding cannot be explained by reciprocal adaptation processes among friends over time.

Overall, most of the significant associations with differences in attitudes relative to parents, friends and classmates were of non-negligible strength, with slightly stronger adaptation processes occurring in relation to friends than to parents and classmates. When parents’ gender beliefs deviated from those of friends and non-befriended classmates, no evidence supporting that peers were more influential than parents was found. Instead, adolescents generally exhibited stronger adaptation when their parents, friends, or classmates were more egalitarian than themselves, whereas adaptations towards more traditional attitudes mainly occurred when both parents and friends or parents and classmates held more traditional views. This suggests that adolescents may tend to adapt to more general norms of increasing egalitarianism and resist such influences only if several relevant socialization agents consistently hold more traditional views.

In line with several previous studies on intergenerational transmission processes of family plans from parents to children (De Valk, 2008; De Valk & Liefbroer, 2007; Sánchez Guerrero & Schober, 2021) as well as on normative influences of classmates (Sánchez Guerrero & Schober, 2021) and friends (van der Vleuten et al., 2019; Wuestenenk et al., 2022), the findings of this study point to a large degree of similarity in the gender socialization influences of parents, friends and classmates across the four European countries. Only for the Netherlands slightly weaker adaptation processes to differences with friends’ attitudes towards the gender division of labor were found. This great similarity across the four countries is remarkable given that they are characterized by substantial variation in gender cultures, which were also observable in the significant differences in starting levels of egalitarianism across countries in our data. Sweden, the country with the most egalitarian gender culture, stood out insofar as Swedish adolescents held the most egalitarian beliefs from the start and did not develop significantly more egalitarian attitudes across the observation period as in the other three countries. This is likely due to a ceiling effect in the attitudes towards the gender division of labor measures, with a significant proportion of Swedish adolescents reporting very egalitarian beliefs at the top of the scale already at the start of the survey. Future quantitative research would benefit from using a larger set of attitudes measures with sufficient variation to capture different types of egalitarianism and multiple dimensions of gender beliefs (Grunow et al., 2018; Knight & Brinton, 2017). The measures of attitudinal similarity or differences between parents and children assume measurement equivalence, which was only partially met. Hence, it cannot be excluded that parents and children interpreted some of the items regarding the division of paid and unpaid work slightly differently. Such differences across generations or life course phases would be an interesting avenue for future psychological research.

The findings regarding the socialization influences of friends and classmates in particular should be treated with some caution. It cannot be excluded that there is potential bias due to reverse causality of adolescents influencing their friends and classmates or that other unobserved factors, such as public and social media, simultaneously affect the gender attitude development of a given adolescent and his/her friends and classmates. These risks are somewhat reduced by focusing on within-individual changes in attitudes towards the gender division of labor as the dependent variable and measuring the differences between adolescents and their parents, friends, and classmates in the previous wave. Further robustness tests using the attitudes of friends’ parents as instrumental variables for friends’ attitudes towards the gender division of labor supported the findings with respect to adaptation to more egalitarian friends. Based on these analyses, adolescents, however, did not seem to adapt significantly to more traditional attitudes held by their friends, which may point to potential biases in this relationship in the OLS models. By examining how within-person changes in friends’ attitudes towards the gender division of labor relate to within-person changes in adolescents’ own attitudes towards the gender division of labor, all time-invariant contextual factors are controlled for, but not the fact that the influence is likely to be bidirectional. Future studies should further address these causality concerns by using longitudinal social network modeling. It was not possible to conduct such analyses with these data while also drawing on the smaller sample of students with parental survey information because our data was not stable enough across the two waves.

Another limitation relates to the use of only binary self-reported measures of gender identity, which are not well suited for capturing the increasing gender fluidity and diversity in individuals’ identities and experiences and in the identity composition of friendship groups and classes (Hyde et al., 2019; Risman, 2017). Lastly, in this study it was not possible to examine more detailed mechanisms underlying gender socialization processes within families, schools, and the classroom. To improve the conceptual understanding and inform possible interventions, it would be important for future research to explore more detailed mechanisms, such as the importance of observing gendered practices, sanctions and rewards, role modeling as well as direct discussions among friends and family members (Bussey & Bandura, 1999). Given that the sample represents youth from four wealthy European countries born around 1996/97, the findings of this study cannot be generalized to other countries and may vary across contexts and time periods with different gender cultures.

## Conclusion

Adolescence is a particularly dynamic period within the life course in terms of value change in which many socialization agents contribute to adolescent´s gender attitude development. However, the joint influence of these actors is rarely studied. This study extends existing research by considering parents, friends, and classmates jointly as gender socialization agents. The analysis of longitudinal data with representative samples of young people aged 15 to 16 years old in Germany, England, Sweden, and the Netherlands suggests that adolescents adapt their attitudes towards the gender division of paid and unpaid work to those of parents, friends, and classmates. This points to the continuing relevance of parents as socialization agents in adolescence, while at the same time underlining the importance of peer groups. The findings on the embeddedness of attitudinal development in different contextual settings, such as families, friendship groups, and classrooms, align well with the multi-layered conceptualization of gender as a social structure. The great similarity of these adaptation processes across countries, gender groups and gender compositions of friendship groups and classes represents an important finding and suggests that the variation in gender culture in this sample of countries does not impact how adolescents respond to gender socialization influences.

## Supplementary information

Supplementary Appendix
