# Supplementary material for: A Time of Great Change: How Parents, Friends, and Classmates Shape Adolescents’ Attitudes towards the Gender Division of Labor
Source: J Youth Adolesc. 2023 Jul 4;52(9):1811–28. doi: 10.1007/s10964-023-01799-2 (PMC10328873; doi:10.1007/s10964-023-01799-2)
Supplement: Supplementary file 1 — Supplementary Appendix [file 10964_2023_1799_MOESM1_ESM.docx]

**Appendix A**

| Table A1. Model fit indices to test measurement invariance between parents and children | | | |
| --- | --- | --- | --- |
|  | CFI | TLI | RMSEA |
| Model 1: all parameters freely estimated | 0.997 | 0.992 | 0.037 |
| Model 2: factor loadings constrained (metric invariance) | 0.996 | 0.994 | 0.033 |
| Model 3: M2 + intercepts constrained (strong invariance) | 0.944 | 0.993 | 0.034 |
| Model 4: M3 + residuals constrained (strict invariance) | 0.935 | 0.944 | 0.099 |
| Model 5: strict invariance plus factor means | 0.915 | 0.932 | 0.109 |

| Table A2. Control variables for the model in Table 2 | | | | | |  |
| --- | --- | --- | --- | --- | --- | --- |
| Control Variables | Model 1 |  | Model 2 |  | Model 3 | |
| Age at wave 1 | 0.080^*^ |  | 0.033^*^ |  | 0.081^*^ | |
|  | (0.032) |  | (0.022) |  | (0.032) | |
| Difference in age between waves | 0.147^***^ |  | 0.141^***^ |  | 0.139^***^ | |
|  | (0.044) |  | (0.041) |  | (0.053) | |
| Female (Ref. Male) | 0.369^***^ |  | 0.360^***^ |  | 0.369^***^ | |
|  | (0.061) |  | (0.058) |  | (0.062) | |
| Immigrant background (Ref. Native/3^rd^ generation) | -0.042 |  | -0.043 |  | -0.046 | |
|  | (0.041) |  | (0.040) |  | (0.041) | |
| Number of friends with AGDL | 0.038 |  | 0.033 |  | 0.038 | |
|  | (0.022) |  | (0.022) |  | (0.022) | |
| All friends are same-sex (Ref. min.1 other-sex friend) | -0.039 |  | -0.038 |  | -0.038 | |
|  | (0.059) |  | (0.061) |  | (0.057) | |
| Same-sex parent (Ref. other-sex parent) | -0.029 |  | -0.023 |  | -0.025 | |
|  | (0.082) |  | (0.081) |  | (0.082) | |
| Share of same-sex classmates | -0.007^*^ |  | -0.007^*^ |  | -0.007^*^ | |
|  | (0.003) |  | (0.003) |  | (0.003) | |
| Parent educational level (Ref. below upper secondary) |  |  |  |  |  | |
| Upper secondary | -0.029 |  | -0.038 |  | -0.026 | |
|  | (0.047) |  | (0.045) |  | (0.048) | |
| University degree | 0.054 |  | 0.054 |  | 0.056 | |
|  | (0.040) |  | (0.040) |  | (0.041) | |
| Mother employed at wave 1 (ref. not employed) | -0.058 |  | -0.060 |  | 0.056 | |
|  | (0.051) |  | (0.051) |  | (0.053) | |
| Religious denomination (Ref. No religion) |  |  |  |  |  | |
| Non-Muslim | -0.098 |  | -0.095 |  | -0.104 | |
|  | (0.068) |  | (0.063) |  | (0.066) | |
| Muslim | -0.201 |  | -0.189 |  | -0.212* | |
|  | (0.104) |  | (0.098) |  | (0.102) | |
| Religiosity | 0.022 |  | 0.019 |  | 0.026 | |
|  | (0.034) |  | (0.032) |  | (0.035) | |
| Share of immigrants in school (Ref. 0-10%) |  |  |  |  |  | |
| 10-30% | 0.029 |  | 0.032 |  | 0.031 | |
|  | (0.036) |  | (0.036) |  | (0.036) | |
| 30-60% | 0.009 |  | 0.016 |  | 0.010 | |
|  | (0.053) |  | (0.052) |  | (0.053) | |
| 60-100% | 0.148^*^ |  | 0.148^*^ |  | 0.143 | |
|  | (0.074) |  | (0.074) |  | (0.073) | |
| Independent schools | -0.181 |  | -0.174 |  | -0.174 | |
|  | (0.130) |  | (0.133) |  | (0.133) | |
| p-values: * <0.05, **<0.01, *** <0.001 |  |  |  |  |  | |

| Table A3. Control variables for the model in Table 3 | | | | | |  |
| --- | --- | --- | --- | --- | --- | --- |
| Control Variables | Model 1 | Model 2 | Model 3 | Model 4 |  | |
| Age at wave 1 | 0.071^*^ | 0.079^*^ | 0.076^*^ | 0.081^*^ |  | |
|  | (0.029) | (0.031) | (0.031) | (0.032) |  | |
| Difference in age between waves | 0.138^***^ | 0.145^***^ | 0.143^***^ | 0.149^***^ |  | |
|  | (0.040) | (0.042) | (0.042) | (0.043) |  | |
| Female (Ref. Male) | 0.219^***^ | 0.275^**^ | 0.307^**^ | 0.314^***^ |  | |
|  | 0.049 | (0.107) | (0.118) | (0.083) |  | |
| Immigrant background (Ref. Native/3rd generation) | -0.041 | -0.044 | 0.041 | -0.041 |  | |
|  | (0.040) | (0.040) | (0.041) | (0.041) |  | |
| Number of friends with AGDL | 0.035 | 0.038 | 0.042 | 0.038 |  | |
|  | (0.021) | (0.022) | (0.022) | (0.022) |  | |
| All friends are same-sex (Ref. min. 1 other-sex friend) | -0.039 | -0.039 | -0.031 | -0.037 |  | |
|  | (0.057) | (0.060) | (0.057) | (0.059) |  | |
| Same-sex parent (Ref. other-sex parent) | -0.022 | -0.029 | -0.024 | -0.028 |  | |
|  | (0.075) | (0.081) | (0.080) | (0.080) |  | |
| Share of same sex classmates | -0.007^*^ | -0.007^*^ | -0.008^*^ | -0.007^*^ |  | |
|  | (0.003) | (0.003) | (0.003) | (0.003) |  | |
| Parent educational level (Ref. below upper secondary) |  |  |  |  |  | |
| Upper secondary | -0.032 | -0.029 | -0.034 | -0.030 |  | |
|  | (0.046) | (0.047) | (0.045) | (0.047) |  | |
| University degree | 0.049 | 0.057 | 0.051 | 0.055 |  | |
|  | (0.039) | (0.040) | (0.039) | (0.040) |  | |
| Mother employed at wave 1 (Ref. not employed) | -0.049 | -0.061 | -0.062 | 0.058 |  | |
|  | (0.054) | (0.050) | (0.050) | (0.052) |  | |
| Religious denomination (Ref. No religion) |  |  |  |  |  | |
| Non-Muslim | -0.097 | -0.093 | -0.095 | -0.097 |  | |
|  | (0.067) | (0.066) | (0.066) | (0.068) |  | |
| Muslim | -0.201 | -0.198 | -0.201 | -0.202 |  | |
|  | (0.103) | (0.103) | (0.104) | (0.105) |  | |
| Religiosity | 0.017 | 0.019 | 0.019 | 0.021 |  | |
|  | (0.033) | (0.033) | (0.035) | (0.034) |  | |
| Share of immigrants in school (Ref. 0-10%) |  |  |  |  |  | |
| 10-30% | 0.027 | 0.027 | 0.027 | 0.029 |  | |
|  | (0.036) | (0.036) | (0.036) | (0.036) |  | |
| 30-60% | 0.002 | 0.008 | 0.007 | 0.009 |  | |
|  | (0.053) | (0.052) | (0.052) | (0.053) |  | |
| 60-100% | 0.143 | 0.147^*^ | 0.142 | 0.153^*^ |  | |
|  | (0.073) | (0.073) | (0.073) | (0.073) |  | |
| Independent schools | -0.183 | -0.187 | -0.186 | -0.181 |  | |
|  | (0.130) | (0.128) | (0.129) | (0.129) |  | |
| p-values: * <0.05, **<0.01, *** <0.001 |  |  |  |  |  | |

Table A4. OLS regression on change in adolescents’ AGDL, interactions with country

| Variables | Model 1 | Model 2 | Model 3 | Model 4 |
| --- | --- | --- | --- | --- |
| AGDL difference with parents (Ref. Similar) |  |  |  |  |
| Adolescent is more egalitarian than parent | -0.368** | -0.260*** | -0.254*** | -0.258*** |
|  | (0.117) | (0.057) | (0.060) | (0.063) |
| Parent is more egalitarian than adolescent | 0.127 | 0.153** | 0.156** | 0.150* |
|  | (0.078) | (0.055) | (0.054) | (0.060) |
| AGDL difference with friends (Ref. Similar) |  |  |  |  |
| Adolescent is more egalitarian than friends | -0.236*** | -0.108 | -0.242*** | -0.225*** |
|  | (0.065) | (0.073) | (0.065) | (0.062) |
| Friends are more egalitarian than adolescent | 0.338*** | 0.466*** | 0.332*** | 0.337*** |
|  | (0.053) | (0.102) | (0.051) | (0.051) |
| AGDL difference with classmates (Ref. Similar) |  |  |  |  |
| Adolescent is more egalitarian than classmates | -0.179** | -0.152** | -0.176** | -0.060 |
|  | (0.066) | (0.057) | (0.062) | (0.072) |
| Classmates are more egalitarian than adolescent | 0.251*** | 0.255*** | 0.249** | 0.342** |
|  | (0.078) | (0.071) | (0.079) | (0.109) |
| Change in friend’s AGDL (Ref. No change) |  |  |  |  |
| Friend group becomes more traditional | 0.029 | 0.019 | 0.019 | 0.031 |
|  | (0.068) | (0.071) | (0.078) | (0.072) |
| Friend group becomes less traditional | 0.097* | 0.089* | 0.102 | 0.098* |
|  | (0.044) | (0.044) | (0.065) | (0.046) |
| Female (Ref. Male) | 0.371*** | 0.378*** | 0.371*** | 0.373*** |
|  | (0.062) | (0.060) | (0.041) | (0.060) |
| Immigrant background (ref. Native/3^rd^ generation) | -0.040 | -0.040 | -0.040 | -0.038 |
|  | (0.041) | (0.042) | (0.041) | (0.041) |
| Country of residence (ref. Sweden) |  |  |  |  |
| England | 0.121 | 0.156 | 0.212* | 0.217 |
|  | (0.068) | (0.108) | (0.085) | (0.180) |
| Germany | 0.066 | 0.133 | 0.039 | 0.214* |
|  | (0.062) | (0.095) | (0.083) | (0.105) |
| The Netherlands | 0.162 | 0.470*** | 0.168 | 0.344 |
|  | (0.120) | (0.102) | (0.137) | (0.183) |

p-values: * <0.05, **<0.01, *** <0.001

|  | |
| --- | --- |
|  |  |

(Table A4 continues on next page)

| *Table A4. Continues* | | | | |
| --- | --- | --- | --- | --- |
| Variables | Model 1 | Model 2 | Model 3 | Model 4 |
| Country * difference with parent (Ref. Sweden / Similar) |  |  |  |  |
| Parent is more traditional & England | 0.190 |  |  |  |
|  | (0.158) |  |  |  |
| Parent is more egalitarian & England | -0.065 |  |  |  |
|  | (0.140) |  |  |  |
| Parent is more traditional & Germany | 0.227 |  |  |  |
|  | (0.129) |  |  |  |
| Parent is more egalitarian & Germany | 0.088 |  |  |  |
|  | (0.093) |  |  |  |
| Parent is more traditional & The Netherlands | 0.005 |  |  |  |
|  | (0.175) |  |  |  |
| Parent is more egalitarian & The Netherlands | 0.019 |  |  |  |
|  | 0.130 |  |  |  |
| Country * difference with friends (Ref. Sweden/ Similar) |  |  |  |  |
| Friends are more traditional & England |  | -0.018 |  |  |
|  |  | (0.122) |  |  |
| Friends are more egalitarian & England |  | -0.128 |  |  |
|  |  | (0.126) |  |  |
| Friends are more traditional & Germany |  | -0.006 |  |  |
|  |  | (0.111) |  |  |
| Friends are more egalitarian & Germany |  | 0.057 |  |  |
|  |  | (0.111) |  |  |
| Friends are more traditional & The Netherlands |  | -0.514*** |  |  |
|  |  | (0.102) |  |  |
| Friends are more egalitarian & The Netherlands |  | -0.338*** |  |  |
|  |  | (0.105) |  |  |
| Country * change in friend's AGDL (Ref. Sweden/ No change) |  |  |  |  |
| Friends become more traditional & England |  |  | -0.173 |  |
|  |  |  | (0.145) |  |
| Friends become more egalitarian & England |  |  | -0.070 |  |
|  |  |  | (0.116) |  |
| Friends become more traditional & Germany |  |  | 0.148 |  |
|  |  |  | (0.120) |  |
| Friends becomes more egalitarian & Germany |  |  | 0.095 |  |
|  |  |  | (0.093) |  |
| Friends becomes more traditional & The Netherlands |  |  | 0.005 |  |
|  |  |  | (0.200) |  |
| Friends becomes more egalitarian & The Netherlands |  |  | -0.054 |  |
|  |  |  | (0.147) |  |

p-values: * <0.05, **<0.01, *** <0.001

(Table A3 continues on next page)

| *Table A4. Continues* | | | | | | |
| --- | --- | --- | --- | --- | --- | --- |
| Variables | | Model 1 | Model 2 | Model 3 | Model 4 |  |
| Country * difference with classmates (Ref. Sweden/ Similar) | |  |  |  |  |  |
| Classmates are more traditional & England | |  |  |  | -0.078 |  |
|  | |  |  |  | (0.187) |  |
| Classmates are more egalitarian & England | |  |  |  | -0.143 |  |
|  | |  |  |  | (0.208) |  |
| Classmates are more traditional & Germany | |  |  |  | -0.116 |  |
|  | |  |  |  | (0.109) |  |
| Classmates are more egalitarian & Germany | |  |  |  | -0.074 |  |
|  | |  |  |  | (0.119) |  |
| Classmates are more traditional & The Netherlands | |  |  |  | -0.289 |  |
|  | |  |  |  | 0.173 |  |
| Classmates are more egalitarian & The Netherlands | |  |  |  | -0.175 |  |
|  | |  |  |  | (0.202) |  |
| Constant | | -1.609** | -1.739** | -1.626** | -1.725*** |  |
|  | | (0.530) | (0.562) | (0.519) | (0.528) |  |
| R2 | | 0.29 | 0.29 | 0.29 | 0.29 |  |
| Note: All models include additional controls for number of friends, whether all friends are of the same sex, whether responding parent is of the same sex, share of same-sex classmates (mean centered), parental education, maternal employment status, religious denomination, religiosity, and share of immigrants in school. | | | | | |  |
| p-values: * <0.05, **<0.01, *** <0.001 |  |  |  |  |  |  |

| Table A5. Correlation matrix among main variables | | | | | | |  |  |  |  |  |  |
| --- | --- | --- | --- | --- | --- | --- | --- | --- | --- | --- | --- | --- |
| Variables | Change  AGDL | | AGDL at wave 1 | AGDL at wave 2 | Difference with parents | Difference with friends | | Change in friend's AGDL | | Difference with classmates | |  |
| Overall (both sexes) |  |  | |  |  | |  | |  | |  | |
| Change in AGDL | 1 |  | |  |  | |  | |  | |  | |
| AGDL at wave 1 | -0.513*** | 1 | |  |  | |  | |  | |  | |
| AGDL at wave 2 | 0.441*** | 0.544*** | | 1 |  | |  | |  | |  | |
| Difference with parents | 0.351*** | -0.500*** | | -0.183*** | 1 | |  | |  | |  | |
| Difference with friends | 0.436*** | -0.750*** | | -0.353*** | 0.414*** | | 1 | |  | |  | |
| Change in friend's AGDL | 0.013 | -0.012 | | 0.002 | -0.034*** | | -0.116*** | | 1 | |  | |
| Difference with classmates | 0.444*** | -0.864*** | | -0.468** | 0.454*** | | 0.734*** | | -0.0159 | | 1 | |
| Male |  |  | |  |  | |  | |  | |  | |
| Change in AGDL | 1 |  | |  |  | |  | |  | |  | |
| AGDL at wave 1 | -0.504*** |  | |  |  | |  | |  | |  | |
| AGDL at wave 2 | 0.476*** | 0.520*** | | 1 |  | |  | |  | |  | |
| Difference with parents | 0.328* | -0.384*** | | -0.375*** | 1 | |  | |  | |  | |
| Difference with friends | 0.415*** | -0.774*** | | -0.377*** | 0.456*** | |  | |  | |  | |
| Change in friend's AGDL | 0.049*** | -0.056*** | | -0.0130 | 0.001 | | -0.075*** | | 1 | |  | |
| Difference with classmates | 0.440*** | -0.864*** | | -0.440*** | 0.494*** | | 0.751*** | | 0.0232 | | 1 | |
| Female |  |  | |  |  | |  | |  | |  | |
| Change in AGDL | 1 |  | |  |  | |  | |  | |  | |
| AGDL at wave 1 | -0.558*** | 1 | |  |  | |  | |  | |  | |
| AGDL at wave 2 | 0.358*** | 0.576*** | | 1 |  | |  | |  | |  | |
| Difference with parents | 0.073*** | -0.321*** | | -0.278*** | 1 | |  | |  | |  | |
| Difference with friends | 0.457*** | -0.730*** | | -0.365*** | 0.402*** | | 1 | |  | |  | |
| Change in friend's AGDL | 0.003 | -0.040*** | | -0.040*** | 0.012 | | -0.146*** | | 1 | |  | |
| Difference with classmates | 0.493*** | -0.865*** | | -0.487*** | 0.433*** | | 0.716*** | | 0.015 | | 1 | |

p-values: * <0.05, **<0.01, *** <0.001

| **Table A6.** OLS regression on change in adolescents' attitudes towards the gender division of labor (AGDL) by country | | | | | | | | | | |
| --- | --- | --- | --- | --- | --- | --- | --- | --- | --- | --- |
|  | England | | Germany | | Netherlands | | | Sweden | | |
| Variables | Cross-country factor ^a^ | Country factor ^b^ | Cross-country factor ^a^ | Country factor ^b^ | Cross-country factor ^a^ | Country factor ^b^ | Cross-country factor ^a^ | | Country factor ^b^ |  |
| AGDL difference with parents (Ref. Similar) |  |  |  |  |  |  |  | |  |  |
| Child is more egalitarian than parent | -0.188 | -0.215* | -0.141* | -0.150** | -0.396** | -0.385** | -0.420*** | | -0.391*** |  |
|  | (0.104) | (0.105) | (0.057) | (0.057) | (0.128) | (0.129) | (0.115) | | (0.107) |  |
| Parent is more egalitarian than the child | 0.109 | 0.050 | 0.234*** | 0.189* | 0.114 | 0.093 | 0.118 | | 0.151 |  |
|  | (0.132) | (0.126) | (0.070) | (0.073) | (0.091) | (0.103) | (0.145) | | (0.148) |  |
| AGDL difference with friends |  |  |  |  |  |  |  | |  |  |
| Child is more egalitarian than the friends | -0.058 | -0.286 | -0.083 | -0.117 | -0.645*** | -0.539*** | -0.095 | | -0.097 |  |
|  | (0.087) | (0.098) | (0.089) | (0.066) | (0.109) | (0.138) | (0.076) | | (0.084) |  |
| Friends are more egalitarian than the child | 0.363* | 0.445** | 0.326*** | 0.302*** | 0.109 | 0.167 | 0.534** | | 0.506** |  |
|  | (0.142) | (0.150) | (0.078) | (0.074) | (0.099) | (0.123) | (0.164) | | (0.169) |  |
| Change in friend's gender AGDL (Ref. No change) | |  |  |  |  |  |  | |  |  |
| Friend group becomes more traditional | -0.157 | -0.213 | 0.145 | 0.108 | -0.052 | 0.079 | 0.009 | | 0.0189 |  |
|  | (0.117) | (0.144) | (0.110) | (0.121) | (0.178) | (0.154) | (0.080) | | (0.065) |  |
| Friend group becomes less traditional | 0.010 | 0.060 | 0.141 | 0.112 | 0.047 | 0.144 | 0.068 | | 0.046 |  |
|  | (0.100) | (0.121) | (0.075) | (0.086) | (0.128) | (0.213) | (0.068) | | (0.079) |  |
| AGDL difference with classmates (Ref. Similar) |  |  |  |  |  |  |  | |  |  |
| Child is more egalitarian than classmates | -0.273 | -0.306 | -0.199* | -0.171* | -0.192 | -0.294 | -0.059 | | -0.053 |  |
|  | (0.197) | (0.208) | (0.085) | (0.984) | (0.155) | (0.167) | (0.066) | | (0.067) |  |
| Classmates are more egalitarian than child | 0.237 | 0.193 | 0.306*** | 0.381*** | 0.174 | 0.124 | 0.287* | | 0.281* |  |
|  | (0.178) | (0.214) | (0.057) | (0.070) | (0.180) | (0.187) | (0.139) | | (0.138) |  |
| Constant | -1.730 | -1.962 | -0.070 | -0.630 | -2.456 | -2.437 | 3.748 | | 3.660 |  |
|  | (0.988) | (0.093) | (0.054) | (0.506) | (1.271) | (1.240) | (3.263) | | (3.250) |  |
| R2 | 0.268 | 0.286 | 0.315 | 0.321 | 0.352 | 0.345 | 0.262 | | 0.260 |  |
| Sample | 682 | 682 | 1,599 | 1,599 | 1,158 | 1,158 | 1,206 | | 1,206 |  |

Note:^a^ The models with cross-country factors are based on the pooled sample of all four countries. ^b^ The models with country factors are based on factor loadings estimated separately for the respective country. Controls: gender of the parent, gender of the child, share of same-sex classmates, share of immigrants in class, age at wave 1 and wave 2, mother works, highest level of parent's education, religion denomination, religiosity, share of immigrants in school, immigrant generation.

p-values: * <0.05, **<0.01, *** <0.001

| Table A7. IV regression with AGDL of the parents of friends as instrumental variable for friends’ AGDL *(N=4522)* | |
| --- | --- |
| Variables | Model 1 |
| AGDL Difference with parents (Ref. Similar) |  |
| Adolescent is more egalitarian than parent | -0.183*** |
|  | (0.032) |
| Parent is more egalitarian than adolescent | 0.189*** |
|  | (0.040) |
| AGDL Difference with friends (Ref. Similar) |  |
| Adolescent is more egalitarian than friends | 0.043 |
|  | (0.145) |
| Friends are more egalitarian than adolescent | 0.542*** |
|  | (0.142) |
| AGDL Difference with classmates (Ref. Similar) |  |
| Adolescent is more egalitarian than classmates | -0.233*** |
|  | (0.049) |
| Classmates are more egalitarian than adolescent | 0.208*** |
|  | (0.054) |
| Change in friend's AGDL (Ref. No change) |  |
| Friend group becomes more traditional | -0.041 |
|  | (0.040) |
| Friend group becomes less traditional | 0.060 |
|  | (0.038) |
| Age at wave 1 | 0.022 |
|  | (0.021) |
| Change in age | 0.061* |
|  | (0.029) |
| Female (Ref. Male) | 0.280*** |
|  | (0.033) |
| Immigrant background (ref. Native/3rd generation) | -0.049 |
|  | (0.035) |
| Country of residence (ref. Sweden) |  |
| England | -0.081 |
|  | (0.070) |
| Germany | 0.098* |
|  | (0.048) |
| The Netherlands | 0.037 |
|  | (0.051) |
| Constant | -0.664 |
|  | (0.353) |

p-values: * <0.05, **<0.01, *** <0.001

*(Table 7 continues in next page)*

| *Table A7. Continues* | |
| --- | --- |
| Tests | Model 1 |
| Underidentification test (Kleibergen-Paaap) | 157.828*** |
| Weak identification test (Kleibergen-Paap rk Wald F) | 83.705 |
| Summary results of first stage regression |  |
| Partial R-Square of excluded instruments (friends more traditional) | 0.273*** |
| Partial R-Square of excluded instruments (friends more egalitarian) | 0.427*** |
| Anderson-Rubin Wald Test (join significance of endogenous regressors) | |
| F-Test | 29.62*** |
| Chi-Square | 59.73*** |

p-values: * <0.05, **<0.01, *** <0.001
